# High-Throughput Analysis of Lidocaine in Pharmaceutical Formulation by Capillary Zone Electrophoresis Using Multiple Injections in a Single Run

**DOI:** 10.1155/2016/4126810

**Published:** 2016-03-16

**Authors:** Andressa C. Valese, Daniel A. Spudeit, Maressa D. Dolzan, Lizandra C. Bretanha, Luciano Vitali, Gustavo A. Micke

**Affiliations:** ^1^Department of Food Science Technologies, Federal University of Santa Catarina, Florianopolis, SC, Brazil; ^2^Departamento de Química, CFM, UFSC, Campus Universitário, Trindade, CP 476, 88040-900 Florianópolis, SC, Brazil

## Abstract

This paper reports the development of a subminute separation method by capillary zone electrophoresis in an uncoated capillary using multiple injection procedure for the determination of lidocaine in samples of pharmaceutical formulations. The separation was performed in less than a minute leading to doing four injections in a single run. The cathodic electroosmotic flow contributed to reducing the analyses time. The background electrolyte was composed of 20 mmol L^−1^ 2-amino-2-(hydroxymethyl)-1,3-propanediol and 40 mmol L^−1^ 2-(*N*-morpholino)ethanesulfonic acid at pH 6.1. The internal standard used was benzylamine. Separations were performed in a fused uncoated silica capillary (32 cm total length, 23.5 cm effective length, and 50 *μ*m internal diameter) with direct UV detection at 200 nm. Samples and standards were injected hydrodynamically using 40 mbar/3 s interspersed with spacer electrolyte using 40 mbar/7 s. The electrophoretic system was operated under constant voltage of 30 kV with positive polarity on the injection side. The evaluation of some analytical parameters of the method showed good linearity (*r* ^2^ > 0.999), a limit of detection 0.92 mg L^−1^, intermediate precision better than 3.2% (peak area), and recovery in the range of 92–102%.

## 1. Introduction

In recent years, capillary electrophoresis (CE) has gained attention of the international scientific community, as an alternative powerful technique for the separation and analysis of compounds of industrial, pharmaceutical, clinical, and environmental interest [1–3]. Some of the features that make the CE an analytical technique of great interest are its wide applicability, being possible to analyze different kinds of samples, the low cost, small amount of waste generated, small sample demand, versatility and especially simplicity in handling, and the speed of experiments that may perform the separation of many compounds in minutes or even in a few seconds [4]. According to recent papers published, the development of rapid methods using CE could be based on strategies as the use of reduced capillary length, application of high electric field, injection at the short-end of the capillary closest to the detector, use of multiple injections in a single run, or even the combinations of all of these strategies [5–8].

The development of simple and fast analytical methods using CE can be further facilitated when using simulation software to select experimental conditions as buffering capacity and conductivity of the medium, electromigration dispersion (EMD) for the analytes, among others. This allows developing an analytical methodology with minimum number of experiments, which can significantly reduce the time spent on this step as shown in different studies described in the literature using the* PeakMaster* ^®^ software [8–13].

Lidocaine is a drug commonly employed as local anesthetic and antiarrhythmic substance present in many commercially available pharmaceutical formulations [14]. Its monitoring in these samples is performed using several methods as high-performance liquid-chromatography (HPLC) with ultraviolet detection (UV) [14–17], HPLC system with UV detection used with no chromatographic column connected [18], capillary electrophoresis (CE) with mass spectrometry (MS) detection [19], and CE with UV detection [5].

Within the papers cited above only one describes the method development exploring the CE potential to create a fast method to determine lidocaine in environmental samples. Geiser and coauthors [5] show a method for separation of lidocaine in less than a minute as a result of exploration of many possibilities for fast CE separation methods, including application of a high electric field through a reduced capillary, short-end injection, multiple sample injections, and use of a dynamically coated capillary to increase the electroosmotic flow (EOF). The background electrolyte (BGE) used was composed of 100 mM trisphosphate at pH 2.5. Considering an uncoated capillary, the EOF value is practically negligible in this pH and thus does not affect the analysis time of the separation of lidocaine. However, the EOF of an uncoated fused silica capillary is one of the characteristics of the CE that could be explored to reduce the separation time of lidocaine. In this type of capillary, the EOF increases at sigmoidal shape with increasing pH of the BGE [11]. Thus, at pH above 5, the EOF cathodic of an uncoated capillary could aid in reducing the separation time of lidocaine, since the mobility of it is added to EOF mobility, that is, separation on the co-EOF mode.

Given the above, the aim of this study is to develop a fast method by capillary zone electrophoresis (CZE) exploring the strategies of multiple injection in a single run and the co-EOF mode to determine lidocaine in the pharmaceutical formulations and also employ the* PeakMaster* software to select the composition of BGE and evaluate some performance parameters of the developed method to determine lidocaine in the samples.

## 2. Experimental

### 2.1. Chemicals and Solutions

The analytical standard of lidocaine hydrochloride and the reagents 2-amino-2-(hydroxymethyl)-1,3-propanediol (TRIS), 2-(*N*-morpholino)ethanesulfonic acid (MES), and benzylamine were purchased from Sigma-Aldrich (St. Louis, USA). Stock solutions of lidocaine 1000 mg L^−1^ and benzylamine 648 mg L^−1^ (internal standard, IS) were prepared in deionized water and stored in a freezer until they are used. The BGE for analysis of lidocaine in the samples was composed of 20 mmol L^−1^ TRIS and 40 mmol L^−1^ MES at pH 6.1 prepared in deionized water. Deionized water (Milli-Q, Millipore, Bedford, MA, United States) was used to prepare all the solutions.

### 2.2. Instrumentation

All experiments were conducted on CE System (7100 Capillary Electrophoresis System, Agilent Technologies, Palo Alto, USA) equipped with a diode array detector set at 200 nm (direct detection), a temperature control device (set at 25°C), and data treatment software (HP ChemStation^®^ *rev A.06.01*). Standards and samples were introduced at the long-end of the capillary and injected using hydrodynamic pressure according to the following steps: 40 mbar/3 s (sample or standard); 40 mbar/7 s (spacer electrolyte); 40 mbar/3 s (sample or standard); 40 mbar/7 s (spacer electrolyte); 40 mbar/3 s (sample or standard); 40 mbar/7 s (spacer electrolyte); and 40 mbar/3 s (sample or standard). The electrophoretic system was operated under positive polarity on the injection side and constant voltage conditions of 30 kV. For all experiments an uncoated fused silica capillary was used purchased from Polymicro Technologies (Phoenix, United States) measuring 32 cm (23.5 cm effective length) × 50 *μ*m i.d. × 375 *μ*m o.d. Initially, the capillary was conditioned by a pressure flush of 1.0 mol L^−1^ of sodium hydroxide (30 min), water (30 min), and BGE (10 min). Between runs, the capillary was rinsed for 0.5 min with the BGE.

### 2.3. Samples

A total of four different samples of pharmaceutical formulations commercially available containing lidocaine as active pharmaceutical ingredient (API) were analyzed: two samples in the form of liquid and two ointments. Liquid samples were appropriately diluted in deionized water before injections. Ointment samples were previously weighted (~200 mg), solubilized in 100.0 mL of deionized water using volumetric flasks, and then diluted in water before injections. After dilutions, the samples were diluted with IS solution (final concentration 64.8 mg L^−1^) and injected into the capillary electrophoresis system.

## 3. Results and Discussion

### 3.1. Method Optimization

**Figure 1:**
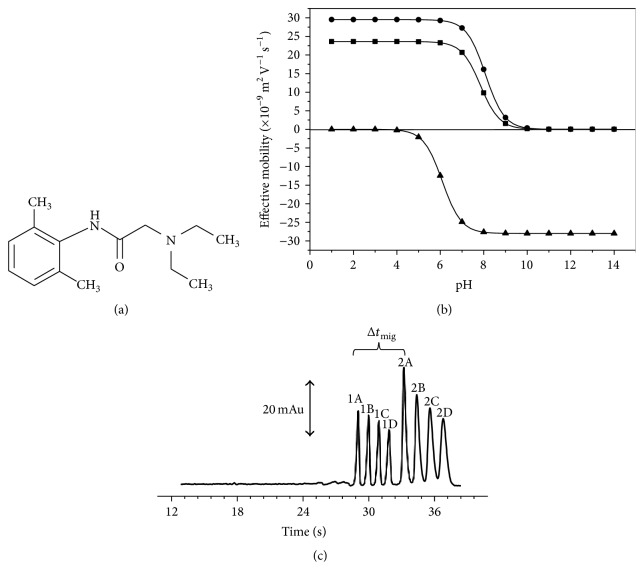
(a) Structure of lidocaine. (b) Effective mobility curves for lidocaine (-■-), TRIS (-●-), and MES (-▲-). (c) Electropherogram of a pharmaceutical formulation using the optimized method by CZE with multiple injections. Peaks: 1: benzylamine (IS); 2: lidocaine; A, B, C, and D represent the first, second, third, and fourth injection, respectively. BGE composed by TRIS 20 mmol L^−1^ and MES 40 mmol L^−1^ at pH 6.1. EOF mobility measured 33.1 × 10^−9^ m^2^ V^−1^ s^−1^.

**Figure 2:**
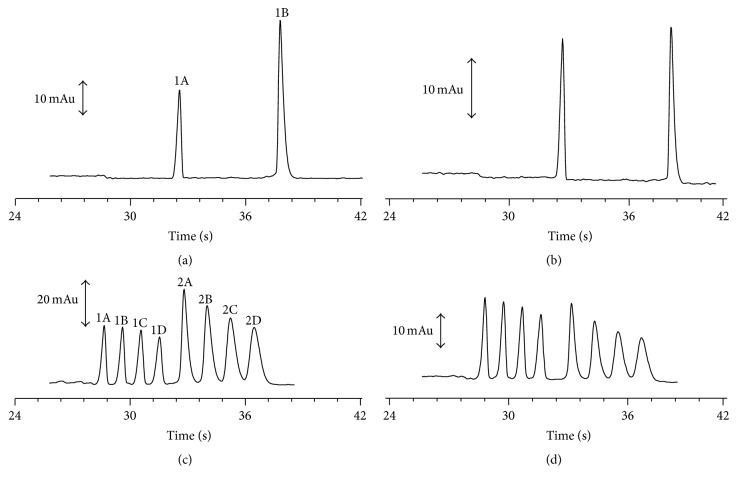
Comparison of single and multiple injections for standard solution ((a) and (c)) and a sample ((b) and (d)). Peaks: 1: benzylamine (IS); 2: lidocaine; A, B, C, and D represent the first, second, third, and fourth injection, respectively.

The development of the method for separation of lidocaine by CZE was based on its structural characteristics (Figure 1(a)). Lidocaine shows chromophores groups that permit employing the direct UV detection mode. Then, the components of BGE should not absorb UV radiation. The selection of pH of the BGE is based on effective mobility* versus* pH curves (Figure 1(b)) constructed using the ion mobility (23.6 × 10^−9^ m^2^ V^−1^ s^−1^) and p*K*_*a*_ (7.85) values of lidocaine obtained from the literature [20]. It is of interest to use a pH equal to or less than 6.5 because lidocaine is highly ionized (cationic form) contributing to a fast separation. However, in a capillary uncoated the EOF is cathodic and increases with the pH of BGE contributing to reducing separation time of lidocaine in the co-EOF mode. Thus, considering the mobility of lidocaine and EOF the pH selected was 6.0. TRIS was selected as the BGE coion since it has an ionic mobility close to the lidocaine ionic mobility (Figure 2(b)), contributing to minimizing its EMD. However, TRIS does not exhibit good buffering capacity at pH around 6. Thus, MES (p*K*_*a*_ 6.15) was selected as counterion because it supplies buffering capacity to the BGE. The optimum concentration of BGE components was TRIS 20 mmol L^−1^ and MES 40 mmol L^−1^. The system parameters predicted by* PeakMaster* software for this BGE were pH 6.0, ionic strength 20 mmol L^−1^, conductivity 0.09 S m^−1^, and buffer capacity 23.5 mmol L^−1^. All these values are appropriate for good characteristics of the CZE method contributing to maintaining constant mobility for lidocaine and EOF along separations. The electromigration dispersion for lidocaine was 1.2 and the internal standard chosen for the method was benzylamine due to its ionic mobility close to lidocaine and absorptivity of UV radiation.

Using the multiple injection procedure is necessary to calculate the sample capacity (*η*_*s*_). For the proposed method the migration time of the fast migrating compound (*t*_mig1_) is of the benzylamine (IS) and its migration time is larger than the time window (Δ*t*_mig_) between the lidocaine and benzylamine. Then, *η*_*s*_ can be calculated employing the following equation [6]:

(1) $$\begin{array}{c} \eta_{s}=\frac{\Delta t_{mig}}{12\sigma }, \end{array}$$

where *σ* represent the broadest peak and can be calculated from the width at 50% of the peak height (*σ* = *w*_50%_/2.35). Using the values of Δ*t*_mig_ 0.1000 min and *w*_50%_ 0.0047 min, *η*_*s*_ calculated was 4.2. Then, as the value obtained was not an integer, we deemed it wise to make the maximum number of injected samples four in a single run. Between each one of the four samples injected spacer electrolyte (40 mBar/7 s) was used for separation of the applied plugs. The experimental electropherogram obtained of a pharmaceutical formulation using the optimized method by CZE with multiple injections is shown in Figure 1(c). It is possible to observe the peaks of the lidocaine and benzylamine related to four samples injected with an extremely short time of separation, less than one minute, in a single run.

### 3.2. Method Figures of Merit

The performance evaluation of the proposed method for determining lidocaine by CZE in pharmaceutical formulations was performed according to ICH guidelines [21]. The parameters analyzed were linearity, limit of detection (LOD) and quantification (LOQ), repeatability (instrumental and intraday), intermediate precision, and selectivity.

The results for parameters evaluated are shown in Table 1. The range of linearity and characteristics of analytical curve were appropriate to lidocaine analysis in samples of pharmaceutical formulations. Furthermore, the LOD and LOQ were less than 3.1 mg L^−1^ allowing the quantification of lidocaine in samples. The precision results were better than 3.2% (intermediate) for peak area and 1.0% (intermediate) for migration time. The recovery experiments were made in three levels of concentration showing range of 92 to 102%, indicating selectivity of the proposed method for lidocaine analysis in the samples. Furthermore, the slopes of the standard addition curve for ointment (0.0184 ± 0.001) and the external calibration curve (0.0186 ± 0.0004) were very close suggesting that the method has no matrix effect for the analyzed samples.

### 3.3. Comparison between Multiple and Single Injections

The multiple injection is a tool that makes it possible to increase the throughput for electrophoretic analysis using a commercial equipment without any modification. However, the effect of multiple injections on the performance of the method should be taken into account. To evaluate these effects on the performance of the proposed methods a sample of pharmaceutical product and a standard solution were injected several times using both single and multiple injections (Figure 2).

By quickly inspecting Figure 2, it is possible to notice that the multiple injections affect the peak shape.

A visual inspection of Figure 2 suggests that the peak shape is affected using the MI mode. This fact could be related to the effective length that is different for each plug injected, leading to difference in the values for the electromigrations dispersion. The fact that the analytes on the last plug will pass through the first plugs of samples and spacers also contributes to the loss in performance when comparing the single and multiple injections. But, on the other hand, these losses in performance are constant and do not affect the quantification parameters (Table 2).

### 3.4. Determination of Lidocaine in Samples

A total of four different pharmaceutical formulations were analyzed using the proposed CZE method with multiple injections and the results are shown in Table 3. The concentrations of the lidocaine determined were close to the nominal concentration for the samples analyzed. The difference between the determined and declared values can be related to several possibilities like bad packaging, exposure to a sun light, and so on, factors that could affect the amount of lidocaine in the sample. The separation was very fast and no interfering peaks from matrix samples were detected. The instrumental throughput of the proposed method was 15 injections per hour using the multiple injection procedure that represents 60 injections per hour (each run has four injections); as a consequence, the analytical frequency using multiple injection mode increases twice comparing to the single injection procedure (30 injections per hour).

## 4. Conclusions

A new fast method to analyze lidocaine by CZE in an uncoated capillary using multiple injections and co-EOF mode was developed. The* PeakMaster* software was an important tool for selecting the BGE composition. The performance parameters evaluated for the proposed method show good results for LOD, LOQ, precision, and recovery indicating that the method is useful for determination of lidocaine in pharmaceutical formulations. The CZE separation obtained with this method, reaching around 60 injections per hour, provides an interesting alternative for routine analysis due to its fast separation, simplicity, and low residue amounts.

## Figures and Tables

**Table 1:** Performance parameters of the method by CZE for analysis of lidocaine.

Parameters	
Number of plates (N m^−1^)	67.000–100.000
Resolution (range for neighboring peaks)	2.00–2.5
Linearity–calibration range (mg L^−1^)^1^	10–50
Linearity–slope (L mg^−1^)^1^	0.0186
Slope standard deviation^1^	3.9 × 10^−4^
Linearity–intercept^1^	0.006
Intercept standard deviation^1^	0.0002
*r* ^1^	0.999
*F*	7858
LOD (mg L^−1^)^2^	0.92
LOQ (mg L^−1^)^2^	3.1
Instrumental precision, RSD (%)–peak area^3^	1.2–2.8 (1.0–3.0)
Instrumental precision, RSD (%)–migration time^3^	0.51–0.55 (0.4–1.3)
Intra-day precision, RSD (%)–peak area^4^	2.7–2.9 (2.5–3.5)
Intra-day precision, RSD (%)–migration time^4^	0.25–1.2 (0.68–1.6)
Intermediate precision, RSD (%)–peak area^4^	2.6–3.2
Intermediate precision, RSD (%)–migration time^4^	0.83–0.95
Recovery (%)^5^, added 8.0 mg L^−1^, found 8.2 mg L^−1^	102
Recovery (%)^5^, added 16.0 mg L^−1^, found 14.7 mg L^−1^	92
Recovery (%)^5^, added 24.0 mg L^−1^, found 23.5 mg L^−1^	98

^1^Six levels of concentration (*n* = 3) genuine replicates, injected in triplicate. ^2^LOD and LOQ calculated using the equations LOD = (3.3 × *s*)/*S* and LOQ = (10 × *s*)/*S*, where *s* is the intercept standard deviation and *S* is the slope of the analytical curve equation, respectively. ^3^Values referents to a standard and a sample, outside and inside the brackets, respectively. RSD range relative to four peaks injected (P1, P2, P3, and P4) (*n* = 12). ^4^Values referents to a standard and a sample, outside and inside the brackets, respectively. RSD range relative to three levels of concentration tested (*n* = 9). ^5^Recovery for an ointment sample.

**Table 2:** Performance comparison for single and multiple injections.

	RSD (%)	
	Multiple injections	Single injections
Peak area^1,2^	0.9–1.5 (1.1–2.4)	0.9 (2.5)
Peak width^1,2^	1.2–2.2 (0.66–1.6)	1.04 (3.4)
Peak high^1,2^	1–2.3 (0.8–1.2)	0.77 (1.8)

^1^Values referents to a standard and a sample, outside and inside the brackets, respectively. RSD range relative to four peaks injected (P1, P2, P3, and P4) (*n* = 10). ^2^Values calculated for lidocaine.

**Table 3:** Lidocaine quantification in the pharmaceutical formulations by CZE method.

Sample	Nominal concentration^1^ (mg)	Found concentration (mg)
(1) Ointment^2^	50	62.8 ± 0.01
(2) Ointment^2^	25	29.19 ± 0.03
(3) Liquid	50	65.58 ± 0.01
(4) Liquid	4	3.70 ± 0.01

^1^Informed by the manufacturer.

^2^mg per gram of ointment.
